# Short uncemented femoral component for hip revision: prognosis and risk factors associated with failure

**DOI:** 10.1186/s12893-021-01196-1

**Published:** 2021-04-13

**Authors:** Zeming Liu, Bo Liu, Bingshi Zhang, Wenhui Ma, Tao Wu, Jia Huo, Sikai Liu, Yongtai Han

**Affiliations:** Department of Orthopaedic Surgery, The Third Hospital of Hebei Medical University, No. 139 Ziqiang Road, Shijiazhuang, Hebei People’s Republic of China

**Keywords:** Hip revision, Short stem, Uncemented

## Abstract

**Background:**

The application of short femoral stems is partially restricted in revision surgery. This study will demonstrate the therapeutic effect and unsuitable situation for short stem revision.

**Methods:**

Demographic characteristics of all patients were recorded in detail (Table 1). Anteroposterior view radiographic examinations of proximal femur are necessary before and after the operation for patients. The primary outcome of interest was the survival rate of the femoral stem at the final follow-up. Risk factors for failure were also investigated. The secondary outcomes of interest included the Harris hip score, excellent to good rate and incidence of complications. The Mann–Whitney U test was performed for comparisons between continuous variables. The chi-square test was performed for comparisons between categorical variables. Cox regression analysis was used to assess the association between potential risk factors and the failure of revision surgery.

**Results:**

A total of 381 patients with short stems were retrospectively reviewed. There were 188 males and 193 females. The average age and body mass index before revision surgery were 58.85 ± 13.46 years and 23.72 ± 3.40 kg/m^2^, respectively. The mid-term survival rate of the short femoral component was 94.23%. The prognosis and complications of patients between the two groups were compared. There was no significant difference between the two groups in the Harris score, complication incidence or survival rate of the femoral component. The strongest risk factor in this study was intraoperative periprosthetic femoral fracture during revision surgery (HR = 5.477, 95% CI = 2.156–13.913).

**Conclusion:**

Three risk factors for failure were identified: ageing, osteoporosis and intraoperative periprosthetic femoral fracture during revision surgery. Therefore, a short femoral stem should be implanted in patients with these risk factors with additional caution.

## Background

Reconstruction of the proximal femur and femoral component implantation is one of the most important processes during hip revision. Traditionally, a lengthened femoral stem could be chosen to achieve stable fixation, which originates from the press fit between the stem and the distal femur [1]. However, there are some disadvantages of this surgical method that might greatly compromise the prognosis of patients. First, due to the physiological curvature of the femur, some lengthened diaphyseal fixation stems have a self-curvature design [2]. The mismatch between the stem and femur might result in implantation difficulty of the stem and intraoperative femoral fracture [3]. Second, if the prognosis of primary revision is not satisfied, secondary revision surgery might be performed. In this situation, the standard stem (total length greater than twice the distance from the tip of the great trochanter to the base with the lesser trochanter vertical distance) and diaphyseal fixation stem used in the first revision are very hard to remove. The bone defects caused by the removal of these femoral stems may make it difficult to implant new femoral prostheses [4, 5]. Finally, the stress shielding effect at the proximal femur could be very strong after implantation of a standard or diaphyseal fixation stem. This issue will lead to bone resorption, osteolysis and bone remodelling of the proximal femur after revision surgery, leaving a large bone deficiency and even causing aseptic loosening of the stem [6]. Therefore, preserving the proximal femoral bone mass is crucial for hip revision.

Recently, the short stem (total length less than twice the tip of the great trochanter to the base of the lesser trochanter vertical distance) [4] has been more commonly chosen in primary arthroplasty. This bone preserved stem is metaphyseal press-fit fixation designed. In theory, the stress shielding effect of proximal femur in patients with short stem implantation will be greatly reduced [7]. For this reason, if a short stem could be used for revision surgery, the proximal femoral bone mass is expected to be increased postoperatively after bone grafting [8]. However, the problem is how these stems achieve early stability without fixation from the distal femur. Furthermore, in patients with serious bone deficiency (e.g., Paprosky Short-B or Type IV), the proximal femur might be completely absent. In this situation, short stems cannot obtain enough press-fit fixation to achieve early stability [9, 10].

The application of a short femoral stem is partially restricted in revision surgery. In some patients with mild to moderate bone deficiency, short stems are certainly a choice. However, the number of reports regarding revision surgery with short femoral stems is limited [11, 12]. We performed hip revisions with short stems in patients with Paprosky Type I, Type II and Type III-A femoral bone deficiency. In this study, these patients were retrospectively reviewed. The follow-up time was at least 5 years. We focused on the prognosis of patients and risk factors for failure. We believe this study will demonstrate the real therapeutic effect as well as the unsuitable situation for short stem revision.

## Methods

### Study population

**Fig. 1:**
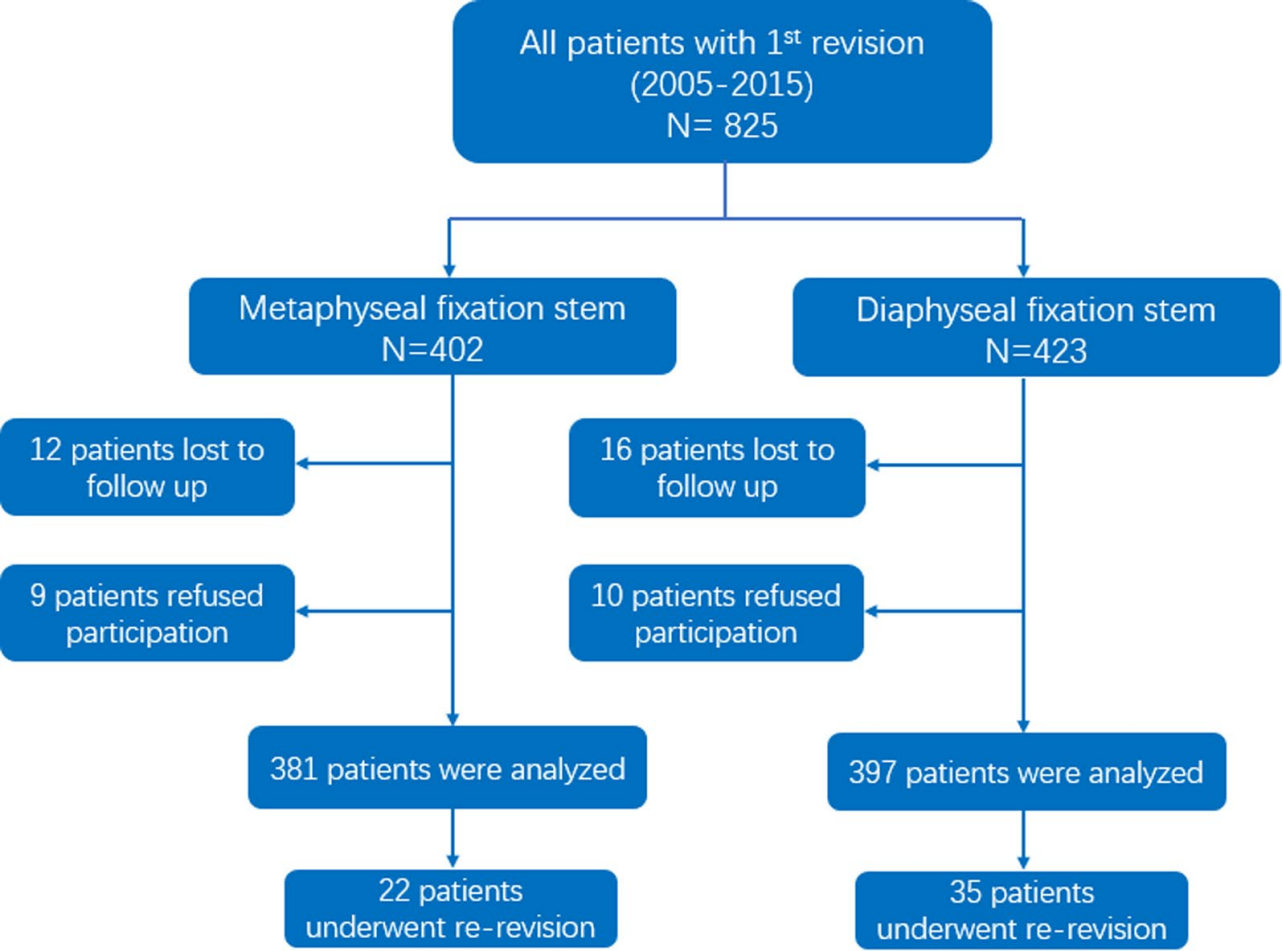
Flowchart for included patients throughout the study

Patients who underwent hip revision from January 2005 to December 2015 were retrospectively analysed in this study (Fig. 1). The inclusion criteria were patients who underwent uncemented hip revision surgery with a short femoral component (in this study, Tri-Lock from DePuy was chosen) and a standard or diaphyseal femoral component (in this study, Wagner SL, Solution, MP and Corail). In the current study, “revision” was defined that the original hip prostheses (the femoral component or spacer) must be removed as well as a new hip prosthesis must be implanted. Patients whose acetabular component was retained but femoral component was replaced were also involved. The exclusion criteria were as follows: (1) revision with cemented femoral component; (2) isolated acetabular component revision failure postoperatively; and (3) loss to follow-up or declined to participate in this study. Demographic characteristics of all patients were recorded in detail (Table 1). Anteroposterior view radiographic examinations of proximal femur are necessary before and after the operation for patients. All demographic and radiological information of patients could be obtained by Picture Archiving and Communication Systems (PACS) in our research institution. The study was approved by the Institutional Review Board of the Third Hospital of Hebei Medical University and was conducted in accordance with the Declaration of Helsinki and regulations of the Health Insurance Portability and Accountability Act (HIPAA). Before the last follow-up time, we obtained written informed consent from patients.

**Table 1:** General information of patients undergoing revision surgery with short femoral components

Patient characteristics	Femoral stem survival (n = 359)	Femoral stem re-revision (n = 22)	Total (n = 381)	Statistical value	P
Gender					
Male	178	10	188	0.141^a^	0.707
Female	181	12	193		
Age (years)	58.15 ± 13.22	70.23 ± 12.45	58.85 ± 13.46	− 4.097^b^	< 0.001
Body mass index (kg/m^2^)	23.72 ± 3.42	23.73 ± 3.18	23.72 ± 3.40	− 0.032^b^	0.975
Smoking					
No	317	20	337	0.138^a^	0.710
Yes	42	2	44		
Alcohol consumption					
No	310	20	330	0.371^a^	0.542
Yes	49	2	51		
Diabetes					
No	320	20	340	0.068^a^	0.795
Yes	39	2	41		
Rheumatism					
No	341	21	362	0.010^a^	0.922
Yes	18	1	19		
Osteoporosis					
No	325	13	338	20.463^a^	< 0.001
Yes	34	9	43		
Indication for revision					
Aseptic loosening	240	16	256	1.522^a^	0.841
Infection	19	2	21		
Recurrent dislocation	73	3	76		
Periprosthetic fracture	25	1	26		
Other	2	0	2		

^a^Chi-square test

^b^Mann–Whitney U test

### Surgical process

**Fig. 2:**
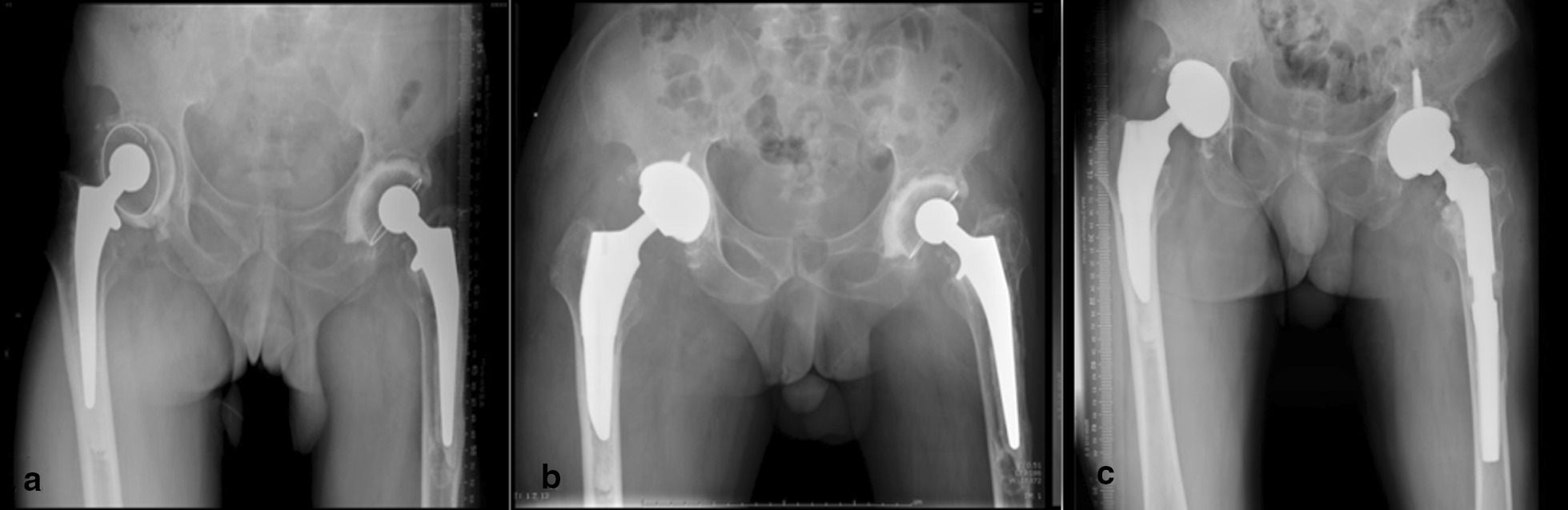
A patient with bilateral hip prostheses aseptically loosened had undergone bilateral primary total hip arthroplasties eight years ago. Cemented prostheses were implanted in both of his hips. Before revision, aseptic loosening of the prostheses could be found in the bilateral hips of the patient. **a** After revision, an uncemented short stem was implanted. **b** At the last follow-up, the prosthesis was stable with satisfactory functional recovery

All revision surgeries with short stems were performed by one group of surgeons. Preoperative radiological images were evaluated to help estimate the bone deficiency around the hip joint. In this study, we focused on femoral bone deficiency. Hence, femoral bone deficiency was classified according to the Paprosky classification system. The surgical process is briefly described as follows. First, the hip joint was clearly exposed via an anterior or posterior approach. The approach was selected according to the preference of the surgeon. During this process, redundant fibroscar tissue was removed. Then, after dislocating the joint prosthesis, the stabilities of both the acetabular component and femoral component were evaluated. The femoral component was removed first to help expose the acetabular component. If the acetabular component was considered to be loosened, it was removed from the bone socket. After acetabular bone grafting, a new acetabular shell and liner were implanted. Next, the proximal femur would be exposed. The surgeon should evaluate the bone deficiency of the proximal femur, which helps to determine the proper kind of femoral stem for revision. In this study, only patients with Paprosky Type I, Type II and Type III A bone deficiency were chosen to implant short femoral stems. If a cemented femoral stem had been used in primary arthroplasty, the bone cement was removed prior to bone grafting and stem implantation. Of course, in some patients, bone cement might not be completely removed. The next step was preparing the medullary canal. Broaches were used to clear the medullary canal. Cancellous bone grafting was performed to help fill the bone deficiency. In some cases, structural bone grafting combined with internal fixation was also used to fill large bone deficiencies. After preparing the medullary canal, the femoral stem and prosthesis head were implanted (Fig. 2). Then, the joint was reduced, and the incision was sutured.

None of the patients were allowed to have weight bearing within the first 3 weeks postoperatively, followed by a gradual increase in partial weight bearing to full weight-bearing 12 weeks after surgery to ensure safety. Anticoagulants were used to prevent deep vein thrombosis.

### Outcomes

The primary outcome of interest was the survival rate of the femoral stem at the final follow-up. Risk factors for failure were also investigated. The secondary outcomes of interest included the Harris hip score, excellent to good rate and incidence of complications. Note that if a revision surgery involving the femoral component was performed in a patient, the last follow-up before secondary revision surgery was considered the final follow-up.

### Statistical analyses

Statistical analyses were performed using SPSS version 19.0 statistical software for Windows (IBM, Armonk, New York). Continuous variables are expressed as the mean ± SD, and categorical variables are expressed as frequencies. The Mann–Whitney U test was performed for comparisons between continuous variables. The chi-square test was performed for comparisons between categorical variables. Cox regression analysis was used to assess the association between potential risk factors and the failure of revision surgery. All variables, including general information, characteristics of primary arthroplasty and characteristics of revision surgical process, were initially included in the regression models. Only the final variables used in the equation for the stepwise regression method are shown. A P value less than 0.05 was considered to be significant.

## Results

### General information

A total of 381 patients with short stems were retrospectively reviewed. There were 188 males and 193 females. The average age and body mass index (BMI) before revision surgery were 58.85 ± 13.46 years and 23.72 ± 3.40 kg/m^2^, respectively. Smoking and alcohol status, comorbidities and osteoporosis status are shown in Table 1. In terms of indication for revision, there were 256 patients with aseptic loosening, 21 patients with infection, 76 patients with recurrent dislocation, 26 patients with periprosthetic fracture and 2 patients with other reasons (one patient with severe thigh pain and one patient with fracture of the ceramic liner). According to the survival status of the femoral component at the final follow-up, patients were divided into two groups (patients with femoral stem survival and patients with femoral stem revision). Comparisons of general information between the two groups are shown in Table 1.

### Surgical characteristics of primary arthroplasty

Primary hip disorders (indication for primary total hip arthroplasty were investigated in this study. Osteonecrosis of the femoral head was the most predominant indication, which consisted of 70% (269/381) of all the individuals. Other indications included avascular necrosis of hip, femoral neck fracture, hip osteoarthritis and development dysplasia of hip. 213 patients underwent cemented fixation and 169 patients underwent uncemented fixation. In terms of the stem fixation segment, the metaphyseal-diaphyseal fixation stem was most commonly chosen during primary total hip arthroplasty (THA). In most situations, a polyethylene liner was implanted. Intraoperative periprosthetic femoral fractures were identified in 35 patients. Before revision surgery, femoral bone deficiency was classified according to the Paprosky classification system. Detailed information regarding the surgical characteristics of primary arthroplasty and femoral bone deficiency is shown in Table 2.

**Table 2:** Primary surgical characteristics and femoral bone deficiency of patients undergoing revision surgery with short femoral components

Patient characteristics	Femoral stem survival (n = 359)	Femoral stem revision (n = 22)	Total (n = 381)	Statistical value	P
Indication for primary THA					
Osteonecrosis	253	16	269	5.130^a^	0.400
Avascular necrosis	20	0	20		
Femoral neck fracture	49	5	54		
Osteoarthritis	24	1	25		
Hip dysplasia	9	0	9		
Other	4	0	4		
Fixation feature of primary THA					
Cemented	200	12	212	0.011^a^	0.915
Uncemented	159	10	169		
Femoral stem fixation segment					
Metaphyseal	21	1	22	2.680^a^	0.262
Metaphyseal-diaphyseal	317	21	338		
Diaphyseal	21	0	21		
Periprosthetic femoral fracture during primary THA					
No	327	19	346	0.554^a^	0.457
Yes	32	3	35		
Bearing					
Metal (ceramic)-polyethylene	319	18	337	1.006^a^	0.316
Ceramic–ceramic	40	4	44		
Femoral bone deficiency (Paprosky classification)					
Type I	186	11	197	0.235^a^	0.889
Type II	149	10	159		
Type-III A	24	1	25		
Short-B	0	0	0		
Type IV	0	0	0		

*THA* total hip arthroplasty

^a^Chi-square test

### Surgical process of revision

Most individuals received their surgeries via a posterior approach. In 76 individuals, bone deficiency was limited after removing the original stem and cement, which allowed new stem implantation without bone grafting. In other patients, bone grafting was performed to reconstruct the bone deficiency. Cancellous bone grafting was independently performed in 268 patients and was combined with structural bone grafting in 37 patients. Intraoperative periprosthetic femoral fractures were identified in 24 patients (8 patients with Vancouver Type A fractures and 16 patients with Vancouver Type B fractures). Because all the femoral stems used in this study were short, in 52 patients, the distal end of the cement was not removed during revision surgery. In 8 patients with periprosthetic femoral fracture during primary THA, the internal fixation was removed during revision surgery. The characteristics of the revision surgical process are summarized in Table 3.

**Table 3:** Characteristics of the revision surgical process of patients undergoing revision surgery with short stems

Characteristics of patients	Femoral stem survival (n = 359)	Femoral stem revision (n = 22)	Total (n = 381)	Statistical value	P
Approach					
Posterior	326	22	348	2.214^a^	0.137
Anterior	33	0	33		
Femoral bone grafting					
None	71	5	76	0.751^a^	0.687
Non-structural	252	16	268		
Structural	36	1	37		
Intraoperative periprosthetic fracture					
No	342	15	357	25.760^a^	< 0.001
Yes	17	7	24		
Residual bone cement					
No (or not applicable)	312	17	329	1.633^a^	0.201
Yes	47	5	52		
Femoral internal fixation remove					
No (or not applicable)	351	22	373	0.501^a^	0.479
Yes	8	0	8		

^a^Chi-square test

### Prognosis and complications

The mean follow-up time was 71.05 ± 16.54 months. Among all 381 surgeries, the femoral component survived in 359 surgeries at the final follow-up. The mean Harris score was 85.36 ± 12.43 at the final follow-up. In 22 patients (5.77%) with poor results, secondary revision surgery was performed to remove the new implanted stem. The average time from primary revision surgery to secondary revision surgery was 16.41 ± 17.47 months (range from 1 to 63 months). The overall excellent-good rate was 80.84%. Complications were identified in 64 patients. The incidence of complications was 16.80%. Postoperative periprosthetic fractures were identified in 9 patients. All these patients experienced hip injures. According to the Vancouver classification system, 6 patients were classified as Vancouver Type A. One patient was classified as Vancouver Type B. This patient received a secondary revision surgery, which replaced the unstable stem by using a diaphyseal fixation stem as well as fixation of the fracture. Two patients were classified as Vancouver Type C. Occasional or recurrent prosthetic dislocations were identified in 20 patients. Superficial surgical site infections were identified in 5 patients. All these infections healed after debridement and wound dressing. In 12 patients, aseptic loosening of the femoral stem was identified. These patients also underwent secondary revision surgery. Mild to moderate (Brooker grade 1–2) heterotopic ossifications were identified in 10 patients. No treatment was taken for these patients. In five patients, signs of bone grafting failure (resorption and osteolysis) were identified. If the stem was loosened after bone grafting failure, a secondary revision surgery was performed to remove the loosened stem and to implant the new lengthened stem. The prognosis and complications of patients in the metaphyseal fixation and diaphyseal fixation stem groups were compared. There was no significant difference between the two groups in the Harris score, complication incidence or survival rate of the femoral component (Table 4).

**Table 4:** Prognosis and complications of patients undergoing revision surgery with short and standard or diaphyseal fixation stems

Patient characteristics	Short stem (N = 381)	Standard and diaphyseal fixation (N = 397)	Statistical value	P
Harris score				
Excellent	149	155	4.583^a^	0.205
Good	159	143		
Fair	51	69		
Poor	22	30		
Complications				
Periprosthetic fracture	9	12	2.197^a^	0.948
Dislocation	16	21		
Recurrent-dislocation	4	5		
Infection	5	6		
Aseptic loosening	12	15		
Heterotopic ossification	10	7		
Bone grafting failure	5	3		
Other	1	1		
Survival of femoral component				
No	359	362	2.707^a^	0.100
Yes	22	35		

^a^Chi-square test

### Risk factors for failure

Three independent risk factors for failure of hip revision using a short femoral stem were identified in this study. Ageing was the first one. In this study, Cox regression analysis revealed that the risk for revision failure increased by approximately 5.6% for every year of age increase (HR = 1.056, 95% CI = 1.012–1.102). Osteoporosis was another independent risk factor for revision failure. Compared with patients without significant osteoporosis, those patients with osteoporosis were 2.8-fold more likely to fail revision surgery with a short femoral stem (HR = 2.802, 95% CI = 1.097–7.157). The strongest risk factor was intraoperative periprosthetic femoral fracture during revision surgery in this study (HR = 5.477, 95% CI = 2.156–13.913). If intraoperative periprosthetic femoral fracture was identified during the revision process, the revision was probably expected to fail in the short term. These independent risk factors, hazard ratios and 95% confidence intervals are shown in Table 5.

**Table 5:** Independent risk factors for early failure of patients undergoing revision surgery with short stems

Risk factors (independent)	Hazard ratio	95% Confidential interval for hazard ratio	P
Age (years)	1.056	1.012–1.102	0.012
Osteoporosis			
No (Ref.)			
Yes	2.802	1.097–7.157	0.031
Intraoperative periprosthetic femoral fracture			
No (Ref.)			
Yes	5.477	2.156–13.913	< 0.001

Only variables in the equation are shown in the table

## Discussion

In this study, the mid-term survival rate of the short femoral component was 94.23%, which was equivalent to what was found in other similar studies. Chatelet et al. [13] reported that the mid-term survival rate was 96.7% for a long uncemented monobloc stem for revision total hip arthroplasty. McInnes et al. [2] also reported similar survival rates of two femoral components for hip revision surgery, which were 87.1% and 87.8% at the 15-year follow-up. This outcome demonstrated the applicability of short stems in patients undergoing hip revision surgery. However, there were still some complications that might compromise the prognosis of patients. The main reason for re-revision was aseptic loosening, followed by bone grafting failure and recurrent dislocation. In patients undergoing hip revision surgery, osteosclerosis and osteolysis can commonly be identified on the proximal femur, especially on the metaphyseal segment [14]. Moreover, loss of cancellous bone would reduce the press-fit effect of the stem. These factors might potentially increase the incidence of aseptic loosening [15]. In addition, allografting was performed in this study. In elderly patients with osteoporosis, bone grafting failure might occur, also causing loosening of the femoral stem [8, 16].

Three risk factors for revision failure were identified in this study. The first one is ageing. As has already been well established, ageing is a certain factor associated with decreasing bone strength, fragility and osteoporosis [17, 18]. Furthermore, osteogenesis is also largely affected by ageing, especially for hip joints. Studies have shown that femoral neck fracture can hardly achieve bony union in elderly patients [19–21]. In patients undergoing hip revision, bone grafting is commonly performed to help fill the bone deficiency of the proximal femur. Meanwhile, impaction bone grafting also plays an important role, which helps stabilize the femoral stem. In elderly patients, graft bone (especially allografts) might not survive. This issue will cause bone resorption around the femoral stem and culminate in aseptic loosening of the stem. In this study, patients were implanted with short stems, which means that bone remodelling largely relies on the success of bone grafting. If bone grafting fails, stem loosening is prone to occur. Some related studies have similar findings. Lamb et al. [12] reported that increasing age (hazard ratio, 1.02 per year) was associated with failure of cemented stem implantation after periprosthetic femoral fracture after primary total hip arthroplasty. Cantrell et al. [22] also found that increasing age was a significant positively associated independent risk factor for the incidence of complications and 30-day readmission. Dale et al. [11] reported that uncemented hip arthroplasties in women aged 55–75 years and over 75 years of age had a higher risk of revision (mainly because of periprosthetic fracture and dislocation) than cemented arthroplasties. Thus, for elderly patients, hip revision with short stems is a delicate problem.

The second risk factor is osteoporosis. In this study, the bone density of the patient was measured by dual-energy X-ray absorptiometry. Because of interference from metal hip prostheses, the bone density of lumbar vertebrae was measured. Patient osteoporosis was diagnosed according to the criteria from World Health Organization (T < − 2.5). Compared to those patients with normal bone mineral density, the risk of revision failure in patients with significant osteoporosis was 2.8-fold higher when a short stem was implanted. The initial stability of uncemented prostheses is dependent on the press-fit between the prosthesis and the bone socket [23]. This means that the minimal anti-rupture strength of the proximal femur must exceed the pressure between the femoral stem and the medullary canal, which is required for stable press-fitting of the prosthesis. In patients with osteoporosis, bone strength decreases, which might cause failure of the press-fit between the prosthesis and medullary canal. Thus, in patients with serious osteoporosis, cemented prostheses, rather than uncemented prostheses, should be implanted. In this study, all patients received uncemented revision, which means that there might be osteolysis, osteosclerosis and bone deficiency around the proximal femur. If the patient has osteoporosis, the incidence of press-fit failure and proximal femoral periprosthetic fracture might be increased, leading to failure of the revision surgery. Furthermore, in patients with osteoporosis, bone grafting might not survive, which could also cause revision failure. Therefore, in patients with osteoporosis, cemented revision should be taken into account.

**Fig. 3:**
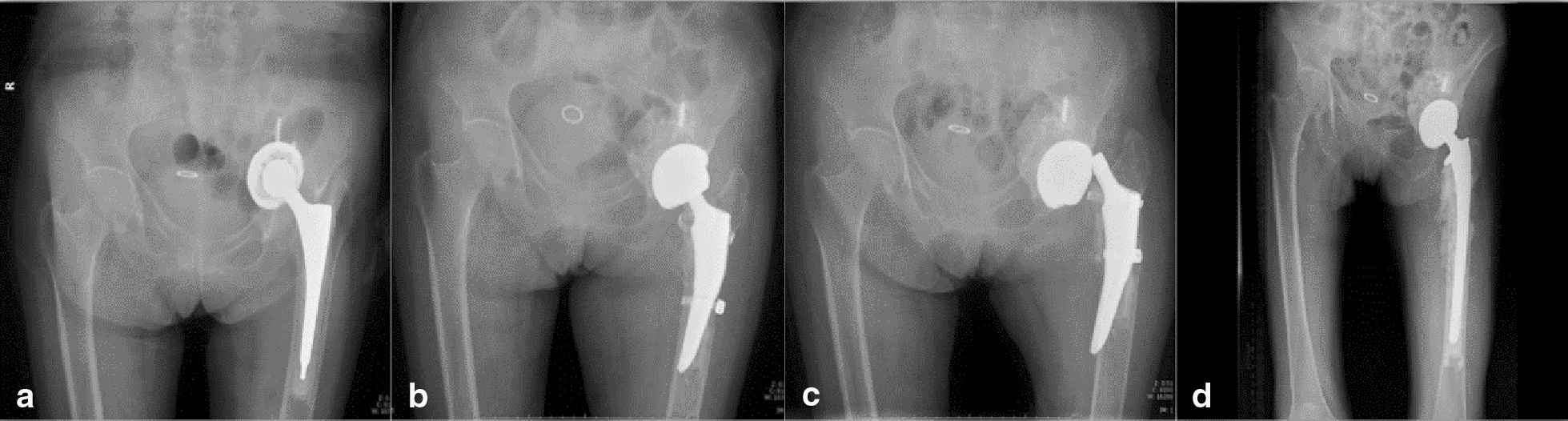
A patient with aseptic loosening of the left hip prosthesis had undergone primary total hip arthroplasty one year prior. An uncemented acetabular component and cemented femoral component were implanted. **a** Before the first revision, an aseptic loosening of the prosthesis could be found. **b** Immediately after the first revision, a periprosthetic femoral fracture could be identified. The fracture was fixed with two cables. **c** Five weeks after the first revision, the stem was loosened. Dislocation between the prosthetic head and femoral stem could also be identified. **d** After the second revision, a lengthened cemented stem was implanted

The strongest risk factor for revision failure in this study was intraoperative periprosthetic femoral fracture during revision surgery. Several reports [24, 25] have shown that, compared to primary hip arthroplasty, hip revision is associated with an increased incidence of periprosthetic femoral fracture. Other studies have shown that compared with the “standard” femoral stem, which is characterized as metaphyseal-diaphyseal fixation, these short stems are commonly associated with an increased incidence of intraoperative periprosthetic femoral fractures. Moreover, periprosthetic femoral fracture is a potential cause of complications. Panula et al. [26] reported that periprosthetic fractures were associated with an increased risk of revision for dislocation after total hip arthroplasty. Devane et al. [27] and Liu et al. [28] also reported that intraoperative periprosthetic femoral fractures were commonly accompanied by poor clinical outcomes of patients. In this study, the femoral component was characterized by metaphyseal fixation and distal end polishing design. This means that the integrity of the local segment of the proximal femur is crucial for the press fit and stability of the stem. Suppose that intraoperative periprosthetic fracture occurs and that the metaphyseal segment of the femur between the greater trochanter and lesser trochanter is involved, there might be not enough press-fit force for stable fixation of the stem. In this situation, the stem is prone to be loosened regardless of internal fixation for periprosthetic fracture. A typical case is shown in Fig. 3. In contrast, if this situation occurs when a “standard” femoral component is implanted, distal press-fit fixation will provide stability for the prosthesis. Therefore, if intraoperative periprosthetic femoral fracture is identified during revision surgery with a short stem, we strongly recommend immediate revision with a long stem (metaphyseal-diaphyseal fixation stem or diaphyseal fixation stem) rather than isolated fracture fixation.

This study has several limitations. First, because of the metaphyseal fixation of short stems, only patients with Paprosky Type I, Type II and Type III A bone deficiency were included in this study. This will make our study unable to be compared with other studies that might involve patients with serious bone deficiency (e.g., Paprosky Short-B or Type IV). Second, isolated acetabular component revision failure postoperatively was excluded from this study. Hence, the overall survival rate of femoral stems might be affected. Third, the sample size was relatively small. Although we added a comparison of different types of femoral stems, a more detailed comparative study will be necessary. Other potential risk factors might exist that were not identified in this study.

## Conclusion

Our study provides detailed information regarding the prognosis of patients undergoing hip revision with a short femoral component. The mid-term results show that the survival rate for the femoral component is 94.23%. Three risk factors for failure were identified: ageing, osteoporosis and intraoperative periprosthetic femoral fracture during revision surgery. Therefore, a short femoral stem should be implanted in these patients with additional caution.

## Data Availability

All data generated or analysed during this study are included in this published article.

## Abbreviations

HIPAA: Health Insurance Portability and Accountability Act
PACS: Picture Archiving and Communication Systems
BMI: Body mass index
THA: Total hip arthroplasty
